# Is visceral adiposity a modifier for the impact of blood pressure on arterial stiffness and albuminuria in patients with type 2 diabetes?

**DOI:** 10.1186/s12933-016-0335-3

**Published:** 2016-01-21

**Authors:** Ryotaro Bouchi, Norihiko Ohara, Masahiro Asakawa, Yujiro Nakano, Takato Takeuchi, Masanori Murakami, Yuriko Sasahara, Mitsuyuki Numasawa, Isao Minami, Hajime Izumiyama, Koshi Hashimoto, Takanobu Yoshimoto, Yoshihiro Ogawa

**Affiliations:** Department of Molecular Endocrinology and Metabolism, Graduate School of Medical and Dental Sciences, Tokyo Medical and Dental University, 1-5-45 Yushima, Bunkyo-ku, Tokyo, 113-8510 Japan; Center for Medical Welfare and Liaison Services, Tokyo Medical and Dental University, Tokyo, Japan; Department of Preemptive Medicine and Metabolism, Graduate School of Medical and Dental Sciences, Tokyo Medical and Dental University, Tokyo, Japan; CREST, Japan Agency for Medical Research and Development, Tokyo, Japan

**Keywords:** Visceral adiposity, Blood pressure, Arterial stiffness, Albuminuria, Type 2 diabetes

## Abstract

**Background:**

We aimed to investigate whether visceral adiposity could modify the impact of blood pressure on arterial stiffness and albuminuria in patients with type 2 diabetes.

**Methods:**

This cross-sectional study examines the interaction of visceral adiposity with increased blood pressure on arterial stiffness and albuminuria. 638 patients with type 2 diabetes (mean age 64 ± 12 years; 40 % female) were enrolled. Visceral fat area (VFA, cm^2^) was assessed by a dual-impedance analyzer, whereby patients were divided into those with VFA < 100 (N = 341) and those with VFA ≥ 100 (N = 297). Albuminuria was measured in a single 24-h urine collection (UAE, mg/day) and brachial-ankle pulse wave velocity (ba-PWV, cm/s) was used for the assessment of arterial stiffening. Linear regression analyses were used to investigate the association of systolic blood pressure (SBP) and VFA with UAE and baPWV.

**Results:**

Patients with VFA ≥ 100 were significantly younger, had higher SBP, HbA1c, triglycerides, UAE, alanine aminotransferase, C-reactive protein and lower high-density lipoprotein and shorter duration of diabetes than those with VFA < 100. SBP was significantly and almost equivalently associated with ba-PWV both in VFA < 100 (standardized β 0.224, p = 0.001) and VFA ≥ 100 (standardized β 0.196, p = 0.004) patients in the multivariate regression analysis adjusting for covariates including age, gender, HbA1c, diabetic complications and the use of insulin and anti-hypertensive agents. By contrast, the association of SBP with UAE was stronger in patients with VFA ≥ 100 (standardized β 0.263, p = 0.001) than that in patients with VFA < 100 (standardized β 0.140, p = 0.080) in the multivariate regression model. In the whole cohort, the significant interaction between SBP and VFA on UAE (standardized β 0.172, p = 0.040) but not on ba-PWV (standardized β −0.008, p = 0.916) was observed.

**Conclusions:**

The effect of increased blood pressure on arterial stiffness is almost similar in type 2 diabetic patients with both low and high visceral adiposity, while its association with albuminuria is stronger in the latter.

## Background

Blood pressure is a strong risk factor for cardiovascular disease (CVD) [1, 2] and chronic kidney disease (CKD) [3–5]. Among patients with diabetes, hypertension is associated with the incidence of CVD and CKD as well [6–9]. The reduction of blood pressure could reduce the risks both for CVD and CKD.

Obesity, especially increased visceral adiposity is a major cause of hypertension, accounting for 65–75 % of the risk for human essential hypertension [10]. In addition, obesity has been reported to be associated with various cardio-metabolic risks including insulin resistance and dyslipidemia, and also be directly associated with CVD [11–14]. Furthermore, abdominal obesity is a strong risk factor for CKD both in general population and patients with diabetes [15, 16]. Therefore, abdominal adiposity is thought to be an important determinant that can account for the association of cardio-metabolic risks with CVD and CKD.

Regarding the association between blood pressure and CVD, the impact of elevated blood pressure on CVD events has been reported to be stronger among people without obesity than those with [17–19]. Also, it has been suggested that normal-weight patients with essential hypertension have increased arterial stiffness [20] and systemic vascular resistance. We recently reported that increased visceral adiposity with normal weight is strongly associated with cardio-metabolic risks and arterial stiffness in patients with type 2 diabetes [21]. These studies imply that visceral adiposity could modify the impact of blood pressure on CVD; however, it is uncertain whether increased blood pressure could more strongly affect arterial stiffening in people with low visceral adiposity than in those with high visceral adiposity. On the other hand, among obese people, especially those with high visceral adiposity, intra-renal renin-angiotensin-aldosterone system is activated [22–24], leading to the glomerular hyperfiltration at the early stage of obesity-hypertension. Hyperglycemia also induces renal damage directly or through hemodynamic modifications including glomerular hyperfiltration [25]. Therefore, it is possible that increase in systemic blood pressure could more strongly affect the renal hemodynamics in obese, especially in obese patients with diabetes, than in non-obese people, resulting in more severe renal manifestations such as increased albuminuria and decreased glomerular filtration rate (GFR). Taken together, we conducted this cross-sectional study to investigate the interaction of visceral adiposity with blood pressure on the increased risk for arterial stiffening and albuminuria in patients with type 2 diabetes.

## Methods

### Subjects

**Fig. 1:**
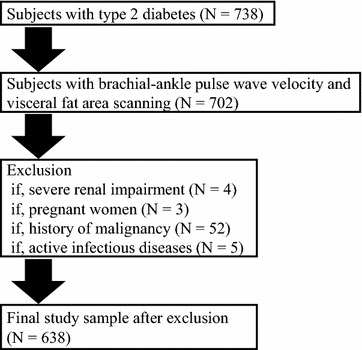
Flowchart of patient recruitment to the study

Patients with type 2 diabetes who admitted to Tokyo Medical and Dental University Hospital for the purpose of glycemic control and/or evaluation of diabetic complications participated in this cross-sectional study. Patients were eligible, if they were aged ≥20 years, and patients who measured both brachia-ankle pulse wave velocity (ba-PWV) and visceral fat area (VFA) and subcutaneous fat area (SFA) by a dual bioelectrical impedance analyzer were enrolled. Patients with severe renal impairment (estimated glomerular filtration rate [eGFR] <15 mL/min/1.73 m^2^ or undergoing renal replacement therapy), pregnant women, and those with infectious or malignant diseases were excluded. Type 2 diabetes was diagnosed according to the criteria of the Japan Diabetes Society (JDS) [26]. As shown in Fig. 1, 638 patients were finally enrolled in this study. This study complies with the principles laid by Declaration of Helsinki and has been approved by the ethical committee of Tokyo Medical and Dental University (No. 1924).

### Clinical and biochemical analysis

Standardized questionnaires were used to obtain information on smoking, medication and past history. Smoking history was classified as either current smoker or non-smoker. CVD was defined as the presence of a previous stroke, myocardial infarction, coronary revascularization procedure. Blood pressure was measured in the sitting position after at least 5 min rest, using an electronic sphygmomanometer (ES-H55, Terumo Inc., Tokyo, Japan). HbA1c was measured by the latex agglutination method. HbA1c levels were expressed in accordance with the National Glycohemoglobin Standardization Programs recommended by the Japanese Diabetes Society [26]. Urinary albumin (UAE) and creatinine excretion were measured by the turbidimetric immunoassay and enzymatic method, respectively, in a single 24-h urine collection. GFR was estimated using the following equation for the Japanese, as proposed by the Japanese Society of Nephrology [27]; GFR = 194 × SCr^−1.094^ × age^−0.287^ [(if female) × 0.739], where SCr stands for serum creatinine in mg/dl, measured by an enzymatic method. Coefficient of variation of R–R intervals (CV-RR) was used for the assessment of diabetic neuropathy. BMI was calculated as weight divided by the square of height (kg/m^2^). VFA and SFA were measured at the level of umbilicus by dual bioelectrical impedance analyzer (DUALSCAN, Omron Healthcare Co., Kyoto, Japan). Patients were divided into those with VFA < 100 cm^2^ (low-V) and those with VFA ≥ 100 cm^2^ (high-V). Brachial-ankle pulse wave velocity (ba-PWV) was measured using a volume-plethysmographic apparatus (BP-203RPE II form PWV/ABI, Omron Healthcare Co., Kyoto, Japan), with subjects in the supine position after at least 5 min of rest [28, 29]. The ba-PWV was calculated as reported previously [30]. We simultaneously measured ba-PWV on both the right and left sides and the averaged values from each individual were subjected to statistical analysis.

### Statistical analysis

Statistical analysis was performed using programs available in the SPSS version 21.0 statistical package (SPSS Inc., Chicago, IL, USA). Data are presented as mean ± SD, median with interquartile range (IQR), or percent as appropriate according to data distribution. Normality was tested by the Kolmogorov–Smirnov test. Differences between low-V and high-V patients were tested with a *t* test or Mann–Whitney U test for continuous variables and Chi square test for categorical variables. Linear regression analyses were used to investigate the association of SBP and VFA with ba-PWV and UAE. We determined the linear relationship and multicollinearity for regression assumptions. We removed one variable if a strong correlation (coefficient of correlation >0.8) was observed between the two independent variables. In order to check the multicollinearity, we evaluated variance infiltration factors. If multicollinearity was found in the data, one variable was removed from the multivariate regression analysis. The following covariates were incorporated into the analysis with a stepwise procedure; duration of diabetes, smoking status, triglycerides, high-density lipoprotein (HDL) cholesterol, low-density lipoprotein (LDL) cholesterol, HbA1c, eGFR, log CRP and the usage of insulin, calcium channel blockers (CCB), angiotensin receptor blockers (ARB), statins and anti-platelet agents. Age and gender were forced into the model. The interaction between SBP and VFA was also investigated in the multiple linear regression analyses. Differences were considered to be statistically significant at p value less than 0.05.

## Results

### Clinical characteristics of patients with low-V and high-V

Among 638 patients, 341 and 297 were classified as low-V and high-V patients. As shown in Table 1, high-V patients were significantly younger, had significantly higher SBP and DBP, lower HDL-C, higher triglycerides levels and a shorter duration of diabetes than the low-V patients. Urinary C-peptide and UAE levels in high-V patients were significantly higher than those in low-V patients. BMI, WC, VFA and SFA levels in high-V patients were significantly higher than in those with low-V. The high-V patients were more frequently receiving CCBs, ARBs and statin therapy and were less likely to receive insulin than low-V patients. baPWV in high-V patients was significantly lower than that in low-V patients.

**Table 1:** Clinical characteristics according to VFA levels

	VFA < 100 cm^2^ (N = 341)	VFA ≥ 100 cm^2^ (N = 297)	p values
Age (years)	66 ± 12	62 ± 13	<0.001
Gender (% male)	57	63	0.196
SBP (mmHg)	128 ± 20	132 ± 17	0.016
DBP (mmHg)	73 ± 12	78 ± 12	<0.001
HbA1c (mmol/mol)	71.6 ± 20.2	75.0 ± 19.5	0.029
HbA1c (%)	8.7 ± 1.8	9.0 ± 1.8	
HDL-cholesterol (mmol/l)	1.32 ± 0.42	1.19 ± 0.31	<0.001
LDL-cholesterol (mmol/l)	2.87 (2.29–3.56)	2.79 (2.26–3.44)	0.515
Triglycerides (mmol/l)	1.31 (0.98–1.86)	1.61 (1.19–2.26)	<0.001
Urinary C-peptide (μg/day)	42 (27–67)	60 (35–99)	<0.001
Duration of diabetes (years)	12 (5–20)	10 (4–16)	0.044
Current smoker (%)	22	25	0.452
History of CVD	13	17	0.183
UAE (mg/day)	11 (7–26)	19 (10–58)	0.001
eGFR (ml/min/1.73 m^2^)	72.0 ± 23.3	71.5 ± 25.6	0.791
AST (U/l)	22 (17–28)	24 (19–41)	<0.001
ALT (U/l)	19 (14–30)	28 (18–48)	<0.001
C-reactive protein (mg/l)	0.80 (0.40–1.95)	1.60 (0.80–3.60)	<0.001
PDR (%)	19	12	0.536
CV-RR (%)	3.3 (2.2–4.8)	3.6 (2.3–5.3)	0.109
ba-PWV (cm/s)	1711 (1459–1906)	1582 (1411–1785)	0.007
Body mass index (kg/m^2^)	23.5 ± 3.2	29.4 ± 4.4	<0.001
Waist circumference (cm)	86 ± 9	102 ± 11	<0.001
Visceral fat area (cm^2^)	74 (57–87)	133 (114–152)	<0.001
Subcutaneous fat area (cm^2^)	144 (120–178)	236 (194–284)	<0.001
Insulin (%)	75	61	0.002
CCBs (%)	29	39	0.023
ARBs (%)	35	53	<0.001
Statin (%)	42	52	0.050
Anti-platelets (%)	17	22	0.322

*ALT* alanine aminotransferase, *ARB* angiotensin receptor blocker, *AST* asparatate aminotransferase, *baPWV* brachial-ankle pulse wave velocity, *CCB* calcium channel blocker, *CVD* cardiovascular disease, *CV-RR* coefficient of variation of R–R intervals, *DBP* diastolic blood pressure, *eGFR* estimated glomerular filtration rate, *HDL* high-density lipoprotein, *LDL* low-density lipoprotein, *PDR* proliferative diabetic retinopathy, *SBP* systolic blood pressure

### Association between SBP and baPWV according to VFA categories

Table 2 shows the linear regression analyses to investigate the association between SBP and ba-PWV in patients with low-V and those with high-V. In the univariate model, SBP was significantly and equivalently associated with ba-PWV. After adjusting for age and gender, the statistical significance of SBP with ba-PWV was unchanged both in patients with low-V and those with high-V. In the multivariate model including covariates such as eGFR and anti-hypertensive agents, the association of SBP with ba-PWV remained significant regardless of visceral adiposity (standardized β 0.224, p = 0.001 in low-V and standardized β 0.196, p = 0.004 in high-V). Among patients with high-V, SFA was inversely associated with ba-PWV (standardized β −0.199, p = 0.007). eGFR was a significant covariate regardless of visceral adiposity.

**Table 2:** Linear regression analysis to investigate the association of blood pressure and visceral adiposity with arterial stiffness in patients with type 2 diabetes

	VFA < 100 cm^2^		VFA ≥ 100 cm^2^	
	Standardized β	p values	Standardized β	p values
Univariate				
Systolic blood pressure	0.183	0.001	0.215	
Age- and gender-adjusted				
Systolic blood pressure	0.172	0.001	0.253	
Age	0.426	<0.001	0.421	
Gender (male versus female)	0.071	0.151	0.044	
Multivariate				
Systolic blood pressure	0.224	0.001	0.196	0.004
Age	0.430	<0.001	0.383	<0.001
Gender (male versus female)	0.130	0.051	0.007	0.920
eGFR	−0.087	0.055	−0.199	0.042
SFA	NA		0.149	0.007
CCB	NA		−0.155	0.031

*CCB* calcium channel blocker, *eGFR* estimated glomerular filtration rate, *SFA* subcutaneous fat area, *VFA* visceral fat area

### Association between SBP and UAE according to VFA categories

Table 3 shows the association between SBP and UAE according to VFA categories among patients with type 2 diabetes. In the univariate model, SBP was significantly associated with UAE both in patients with low-V and those with high-V. The association of SBP with UAE was unchanged in age- and gender-adjusted model regardless of visceral adiposity (standardized β 0.205, p = 0.001 in patients with low-V and standardized β 0.290, p < 0.001 in patients with high-V). In the multivariate model adjusting for covariates including age, gender, diabetic complications such as neuropathy and retinopathy and HbA1c level, SBP remained significantly associated with UAE in patients with high-V (standardized β 0.263, p = 0.001); whereas, its association with UAE was attenuated in those patients with low-V (standardized β 0.140, p = 0.080).

**Table 3:** Linear regression analysis to investigate the association of blood pressure and visceral adiposity with albuminuria in patients with type 2 diabetes

	VFA < 100 cm^2^		VFA ≥ 100 cm^2^	
	Standardized β	p values	Standardized β	p values
Univariate				
Systolic blood pressure	0.203	0.001	0.280	<0.001
Age- and gender-adjusted				
Systolic blood pressure	0.205	0.001	0.290	<0.001
Age	0.079	0.188	0.172	0.172
Gender (male versus female)	0.074	0.219	0.087	0.087
Multivariate				
Systolic blood pressure	0.140	0.080	0.263	0.001
Age	−0.042	0.649	−0.090	0.236
Gender (male versus female)	0.120	0.122	0.166	0.28
eGFR	−0.191	0.042	NA	
Insulin	0.145	0.064	NA	
PDR	0.172	0.024	NA	
CV-RR	−0.142	0.075	−0.161	0.034
HbA1c			0.135	0.076

*CV-RR* Coefficient of variation of RR intervals, *eGFR* estimated glomerular filtration rate, *PDR* proliferative diabetic retinopathy, *VFA* visceral fat area

### Interaction between SBP and VFA accounting for the risk of arterial stiffening and albuminuria

Table 4 shows the multivariate linear regression analyses to investigate whether binary interaction between SBP and VFA could account for the risks of arterial stiffening and albuminuria in the whole cohort. The significant interaction between SBP and VFA was observed in the model where UAE was used for a dependent variable; whereas, no significant interaction of SBP with VFA was found as for ba-PWV.

**Table 4:** Interaction between blood pressure and visceral adiposity accounting for the risk of arterial stiffening and albuminuria in patients with type 2 diabetes

	ba-PWV		UAE	
	Standardized β	p values	Standardized β	p values
SBP × VFA	−0.008	0.916	0.172	0.040
Systolic blood pressure	0.177	<0.001	0.171	0.001
Visceral fat area	0.149	0.149	−0.060	0.471
Age	0.430	<0.001	NA	
Body mass index	−0.299	0.001	NA	
eGFR	−0.146	0.008	NA	
Calcium channel blocker	0.109	0.029	NA	
HbA1c	NA		−0.138	0.009
CV-RR	NA		0.148	0.005
Gender (male versus female)	NA		0.130	0.015
Angiotensin receptor blocker	NA		0.114	0.030
Insulin	NA		0.109	0.035

*ba-PWV* brachial-ankle pulse wave velocity, *CV-RR* Coefficient of variation of RR intervals, *eGFR* estimated glomerular filtration rate, *SBP* systolic blood pressure, *UAE* urinary albumin excretion, *VFA* visceral fat area

## Discussion

Both increased arterial stiffness and albuminuria are strong predictors for mortality, CVD and CKD in patients with diabetes [31–36]. Therefore, it is important to elucidate the high risk groups both for increased arterial stiffness and albuminuria among diabetic patients. This study clearly demonstrates that increased SBP can equivalently account for the risk for arterial stiffening regardless of visceral adiposity; whereas, the impact of SBP on albuminuria is stronger in diabetic patients with high visceral adiposity than those with low visceral adiposity.

### Association of blood pressure and visceral adiposity with organ damage

Visceral adiposity has been reported to be associated with incident hypertension [37, 38] and albuminuria [39, 40]. More recently, we found that high visceral fat with low subcutaneous fat accumulation is an important determinant of carotid atherosclerosis and high subcutaneous fat could be protective against atherosclerosis in patients with type 2 diabetes [41], and others reported that subcutaneous fat thickness assessed by ultrasound is inversely associated with carotid atherosclerosis in diabetic patients, particularly in men [42]. Moreover, visceral adiposity is strongly associated with the alteration of myocardial glucose uptake and its association further relates to type 2 diabetes [43]. These studies suggest that visceral and subcutaneous adiposities are directly associated not only cardio-metabolic risks but also target organ damage including heart and arterial wall injuries. We found in this study a stronger association of blood pressure with albuminuria in patients with high visceral adiposity than those with low visceral adiposity, suggesting that visceral adiposity could modify the association of blood pressure at least with albuminuria in patients with type 2 diabetes.

### Potential mechanisms regarding the interaction between blood pressure and adiposity on albuminuria

By which mechanisms are involved in the greater impact of elevated blood pressure on albuminuria in patients with high visceral adiposity than in those with low visceral adiposity? Sympathetic activity and local (renal) renin-angiotensin-aldosterone system could account for the association. Obesity increases sympathetic activity in the kidneys and skeletal muscle; however, cardiac sympathetic activity may not be elevated [44–46]. Furthermore, excessive weight gain, especially visceral adiposity increases leptin level, promotes renal compression, activates renal renin-angiotensin-aldosterone system [47], all of which could impair renal-pressure natriuresis, increase glomerular pressure, leading to progression of albuminuria. These observations could at least partly explain why elevated blood pressure is more strongly associated with albuminuria among patients with high visceral adiposity than among patients with low visceral adiposity.

### Strengths and limitations

The strength of our study is that we directly measured VFA by a dual-impedance analyzer for the assessment of visceral adiposity. Previous studies assessed the interaction of adiposity with the association between hypertension and CVD using BMI or WC [7, 8, 48]. Thus, to the best our knowledge, this study is the first to investigate the interaction of visceral adiposity directly measured and blood pressure both with arterial stiffness and albuminuria. This study has a couple of limitations that should be mentioned. First, it has recently been reported that absolute loss of visceral fat mass may play a major role in resolution of diabetes following bariatric surgery, regardless of the amount of weight loss [49], suggesting the importance of prospectively evaluating the change in visceral adiposity to investigate the association between cardio-metabolic risks including blood pressure and organ damage such as arterial stiffening and albuminuria; however, it is impossible to infer causality because of its cross-sectional design. Second, population in this study was ethnically and socially homogeneous, because this study was hospital-based; therefore, generalization of our findings might be limited. Third, we were unable to obtain information on renin-angiotensin-aldosterone system and sympathetic activity. Fourth, we were unable to obtain any information on diet including vitamin A which may reduce visceral fat [50]. Finally, it is to be elucidated whether the association of blood pressure with arterial stiffness and albuminuria could be mediated by visceral adiposity in populations other than diabetic patients.

## Conclusion

The effect of increased blood pressure on arterial stiffness is almost similar in type 2 diabetic patients with both low and high visceral adiposity, while its association with albuminuria is stronger in the latter.

## Abbreviations

ALT: Alanine Aminotransferase
ARB: angiotensin receptor blocker
AST: Asparatate Aminotransferase
baPWV: brachial-ankle pulse wave velocity
CCB: calcium channel blocker
CI: confidence interval
CRP: C-reactive protein
CVD: cardiovascular disease
CV-RR: coefficient of variation of R–R intervals
eGFR: estimated glomerular filtration rate
HDL: high-density lipoprotein
LDL: low-density lipoprotein
PDR: proliferative diabetic retinopathy
SFA: subcutaneous fat area
UAE: urinary albumin excretion
VFA: visceral fat area
